# Amorphous Mo_5_O_14_-Type/Carbon Nanocomposite with Enhanced Electrochemical Capability for Lithium-Ion Batteries

**DOI:** 10.3390/nano10010008

**Published:** 2019-12-18

**Authors:** Ahmed M. Hashem, Ashraf E. Abdel-Ghany, Rasha S. El-Tawil, Sylvio Indris, Helmut Ehrenberg, Alain Mauger, Christian M. Julien

**Affiliations:** 1 National Research Centre, Inorganic Chemistry Department, 33 El Bohouth St. (former El Tahrir St.), Dokki, Giza P.O.12622, Egypt; 2 Karlsruhe Institute of Technology (KIT), Institute for Applied Materials (IAM), Hermann-von-Helmholtz-Platz 1, D-76344 Eggenstein-Leopoldshafen, Germany; 3 Institut de Minéralogie, de Physique des Matériaux et de Cosmologie (IMPMC), Campus Pierre et Marie, Sorbonne Université, UMR 7590, 4 place Jussieu, 75005 Paris, France; alain.mauger@upmc.fr

**Keywords:** lithium batteries, anode material, Mo_m_O_3m−1_ oxide, Mo_5_O_14_-type/C composite

## Abstract

An amorphous Mo_m_O_3m−1_/carbon nanocomposite (*m* ≈ 5) is fabricated from a citrate–gel precursor heated at moderate temperature (500 °C) in inert (argon) atmosphere. The as-prepared Mo_5_O_14_-type/C material is compared to α-MoO_3_ synthesized from the same precursor in air. The morphology and microstructure of the as-prepared samples are characterized by scanning electron microscopy (SEM), X-ray diffraction (XRD), and Raman scattering (RS) spectroscopy. Thermal gravimetry and elemental analysis indicate the presence of 25.8 ± 0.2% of carbon in the composite. The SEM images show that Mo_5_O_14_ is immersed inside a honeycomb-like carbon matrix providing high surface area. The RS spectrum of Mo_5_O_14_/C demonstrates an oxygen deficiency in the molybdenum oxide and the presence of a partially graphitized carbon. Outstanding improvement in electrochemical performance is obtained for the Mo_5_O_14_ encapsulated by carbon in comparison with the carbon-free MoO_3_.

## 1. Introduction

Due to their numerous applications including their vital role as light weight, long life and high-energy density power sources, special attention has been directed towards lithium-ion batteries (LIBs). These power sources find major applications from portable electronic appliances to electric vehicles. As the positive electrode plays a major role in the electrochemical performance of the LIBs, many efforts of research are currently made on active electrode materials with high capability and long cycling life [1]. Among the molybdenum-oxide series evaluated as electrode materials, α-MoO_3_ and MoO_2_ are thermodynamically stable phases. They crystallize in a unique layered structure (orthorhombic, *Pbnma* space group) and a rutile-like structure (monoclinic *P*2_1_/*c* space group), respectively [2]. In the same Mo-O binary system, the oxygen deficient Mo_m_O_3m−1_ phases include Mo_4_O_11_, Mo_5_O_14_, Mo_8_O_23_, Mo_9_O_26_, and Mo_17_O_47_, which crystallize in the so-called Magnéli phases described in details by Kihlborg [3]. These oxides have been identified as derived from MoO_3_ by shear mechanism with networks of MoO_6_ octahedra. These units are connected in three dimensions with empty parallel tunnels involving the presence of potential sites for hosting Li^+^ ions. They are performant cathode materials in non-aqueous lithium cells [4,5,6,7,8]. Few works reporting the lithiation of Mo_5_O_14_ are available in the literature [5,9,10,11]. Preliminary patterns of the fully lithiated Mo_5_O_14_ crystal suggest that the unit cell expands in the *b* direction and contracts in both the *a* and *c* directions upon insertion of lithium [9]. Cignini et al. reported a specific capacity of 310 mA g^−1^ for Mo_5_O_14_ as the positive electrode of a primary Li cell discharged at 0.5 mA cm^−2^ and 1.0 V cut-off. [5]. Nazri and Julien [10] showed that MoO_2.8_, an oxygen-deficient Mo_5_O_14_ prepared by dehydration and annealing treatment of molybdic acid powder (MoO_3_·1H_2_O) at 750 °C, exhibited a high electrical conductivity of 10^−2^ S cm^−1^. Such a material displayed an excellent reversible capacity (1.45 Li/Mo) in cells with 1 mol L^−1^ LiClO_4_ in propylene carbonate (PC) electrolyte in the potential range 3.3–1.3 V vs. Li^+^/Li.

However, MoO_3_ and Mo_m_O_3m−1_ lithiated oxides suffer from a large volume expansion (about 104% for MoO_3_). During Li^+^ insertion, pulverization of the cathode occurs at a potential of less than 1.5 V and disconnection from the current collector. As a result, the electrochemical performance is poor [12]. Different strategies can be used to improve the cyclability as follows. Nanosized Mo_m_O_3m−1_ particles were prepared with low crystallinity [13,14]. Blended hybrid was fabricated [15]. Composites with addition of an electronically-conductive carbonaceous materials were synthesized [12,16,17,18,19]. The carbon has two beneficial effects: it opposes the aggregation of the active particles and it accommodates the volume change of electrode material during cycling [17]. Different synthesis techniques were used to fabricate MoO_3_/C composites: electrospinning method [18], hydrothermal route [12,16,19,20], template method [21], spray pyrolysis [22] and ball milling [23].

In this paper, a nanocomposite of oxygen-deficient Mo_m_O_3m−1_/carbon is synthesized by a sol-gel method using citric acid as chelating agent. The calcination of dried precursor in argon atmosphere results in the formation of the amorphous-like Mo_5_O_14_ (MoO_2.8_) oxide immersed in a carbon matrix. This new material is characterized by X-ray diffraction (XRD), scanning electron microscopy (SEM), surface area analysis (BET) and Raman scattering (RS) spectroscopy. Finally, electrochemical properties of MoO_3_/C composite for lithium ion batteries were also investigated for comparison.

## 2. Materials and Methods

**Scheme 1:**
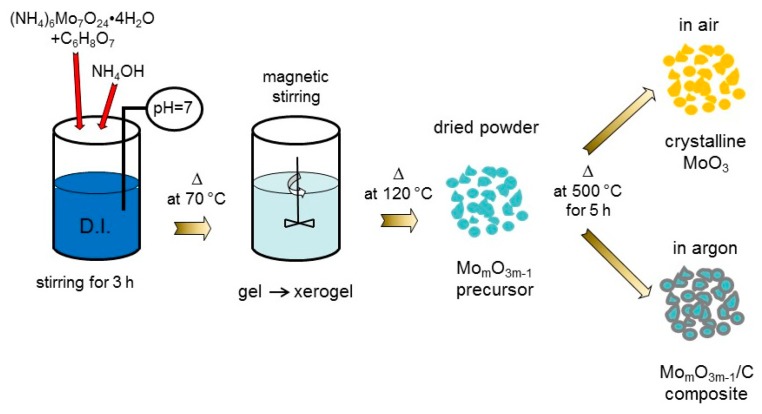
Overall synthesis process of the crystalline MoO_3_ and Mo_m_O_3m−1_/C composite.

Pure MoO_3_ (P-MoO_3_) sample and Mo_m_O_3m−1_/C nanocomposite were prepared by a citrate–gel method using ammonium molybdate tetrahydrate (AMT, (NH_4_)_6_Mo_7_O_24_∙4H_2_O). First, a stoichiometric amount of AMT was dissolved in de-ionized water (DI) and stepwise added to the stirring aqueous solution of citric acid (CA) with metal/CA ratio of 1:1 under neutral pH adjusted with ammonium hydroxide; Second, the overall solution was stirred for 3 h to form homogenous mixture through reaction between the metal ion and the chelating agent. Transparent gel was formed after a slow evaporation of the solution heated at 70 °C. Furthermore, continuous heating with stirring led to the transformation of the gel to xerogel, which converted to powder by further drying at ca. 120 °C. Pure MoO_3_ crystals were obtained by calcining the milled precursor at 500 °C for 5 h in air, while the Mo_m_O_3m−1_/C composite was formed under argon atmosphere. The overall synthesis process is presented in Scheme 1.

The composition was determined by thermal gravimetric analysis (TGA) and differential scanning calorimetry (DSC) using an analyzer model TGA-7 series (Perkin Elmer, Waltham, MA, USA,) in the temperature range of 30–1000 °C in air at a heating rate of 10 °C min^−1^. Elemental analysis was performed using a CHNOS analyzer (Elementar Analysen Systeme GmbH, Langenselbold, Germany). The structure of the samples was analyzed by X-ray diffraction (XRD) using a X’Pert diffractometer (Philips, Hamburg, Germany) equipped with a CuK_α_ X-ray source (λ = 1.54056 Å). Data were collected in the 2*θ* range of 10–80°. The particle morphology was examined by scanning electron microscopy (SEM) (JEOL microscope, Akishima, Japan). Raman spectra were collected at a spectral resolution of 2 cm^−1^ with a double monochromator (Jobin-Yvon model U1000) using the 514.5 nm line of an Ar-ion laser (Spectra-Physics 2020) in a backscattering geometry. The laser power was kept below 25 mW to prevent the sample degradation by the laser spot. The specific surface area was measured by nitrogen adsorption/desorption at 77 K using the Brunauer-Emmett-Teller (BET) method (Quantachrome NOVA Automated Gas Sorption).

Electrochemical properties were investigated by cyclic voltammetry (CV) and galvanostatic charge-discharge (GCD) techniques. The cathodes were prepared by mixing 80% (*w*/*w*) of the active material, 10% (*w*/*w*) super C65 carbon (TIMCAL, Lac-des-Iles, Canada) and 10% (*w*/*w*) polyvinylidenefluoride (Solef PVdF 6020 binder, Solvay, Brussels, Belgium), in N-methyl-2-pyrrolidone (NMP, Sigma-Aldrich, St. Louis, MO, USA) to get a slurry, which was coated on copper foil at a loading of 2.1 mg cm^−2^. After drying over night at 80 °C, discs were punched out of this film with a diameter of 1.4 cm. The 2032-type coin cells were assembled in an argon-filled glove box with lithium foil (Alfa Aesar, Haverhill, MA, USA) as the anode, 1 mol L^−1^ LiPF_6_ in ethylene carbonate:dimethyl carbonate (EC:DMC) = 50:50 (*v*/*v*) (LP30, Sigma-Aldrich, battery grade) as the electrolyte, and glass microfiber filters (Whatmann^®^-GF/D 70 mm Ø) as the separator. A multi-channel potentiostat (VMP3, Bio-Logic, Seyssinet-Pariset, France) was used to test the electrochemical performance of the electrodes at 25 °C in the voltage range of 0.6–3.5 V vs. Li^+^/Li.

## 3. Results

### 3.1. Structure and Morphology

**Figure 1:**
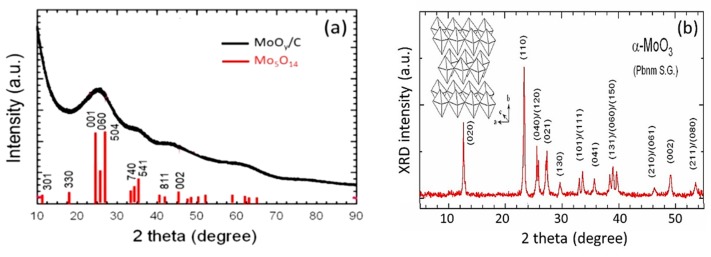
XRD pattern of (**a**) Mo_5_O_14_/C composite compared with the diagram of Mo_5_O_14_ as reference [26] and (**b**) crystalline a-MoO_3_ (orthorhombic structure).

**Figure 2:**
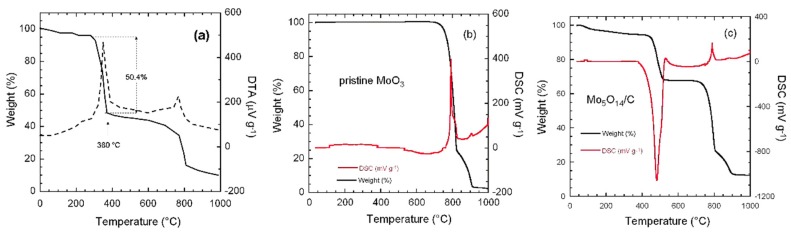
TGA and DSC curves of the MoO_3_ synthesis (**a**) and as prepared P-MoO_3_ (**b**) and Mo_5_O_14_/C composite (**c**) recorded at heating rate of 10 °C min^−^^1^.

Figure 1a shows the XRD pattern of the molybdenum oxide/carbon composite obtained by calcination of ammonium molybdate tetrahydrate in argon atmosphere. This diagram exhibits four broad peaks centered at 2*θ* angles of 25.7, 35.1, 43.5 and 62.2°, which suggest a quasi-amorphous texture of the Mo oxide in a carbon matrix obtained by the decomposition of the chelating agent in absence of oxygen. The pattern shown in Figure 1a compares well to that of the tetragonal Mo_5_O_14_ phase (*P*4/*mbm* space group, *a* = 2.2989 nm, and *c* = 0.3936 nm) reported by Kihlborg [24]. Note this composition deduced form this XRD pattern implies the molybdenum valence distribution Mo^6+^_3_Mo^5+^_2_. This valence mixing in turn implies a high concentration of free carriers and thus a large electrical conductivity, which will be beneficial to the electrochemical properties. The crystal structure of Mo_5_O_14_ can be depicted as complexes of linked MoO_6_ octahedra and MoO_7_ pentagonal bipyramids [25]. The peak at 2*θ* = 25.7° corresponds to the overlapping (001), (060) and (504) lines of the Mo_5_O_14_ lattice, while the less intense peaks at 2*θ* = 35.1° and 43.5° coincide with the (541) and (002) Bragg lines of the tetragonal structure, respectively [26]. The crystallite size calculated by the Scherrer’s formula from the full-width of the broad line at 2*θ* = 25.7° is found to be 2.9 nm. The presence of the disordered carbon (low degree of graphitization) is evidenced by the main signal at 2*θ* ≈ 26° ((002) Bragg line) probably overlapping with the main signals of the Mo_5_O_14_ phase. However, the amorphous nature of carbon deposited on the Mo oxide at a low temperature of 500 °C is confirmed by the absence of the (100) and (101) carbon-related peaks in the 40–45° 2*θ* range [16]. The formation of an amorphous Mo_5_O_14_/C composite under argon gas is due to the presence of CO and CO_2_ gases generated by the combustion reaction of the carbon source using citric acid, which reduces the Mo^6+^ ions of the ammonium molybdate. The TGA experiments performed during the synthesis (Figure 2a) present a strong exothermic peak at ca. 380 °C, which corresponds to the combustion of the organic compound C_6_H_8_O_7_ accompanied by the decomposition of (NH_4_)_6_Mo_7_O_24_ (partial desorption of H_2_O and NH_3_) into oxides. At this stage, a weight loss of almost 50% was recorded.

The XRD pattern of P-MoO_3_ (Figure 1b) displays the typical pattern of a well-crystallized α-MoO_3_ phase that can be indexed in the orthorhombic *Pbnm* space group (JPCDS card 76-1003). The coarse-grained powder presents a preferred orientation of (0k0) planes, as the (020), (040) and (060) Bragg lines have a large intensity. Rietveld refinement provided lattice constants of *a* = 0.3965 Å, *b* = 1.3861 Å, and *c* = 0.3697 Å.

The carbon content of the Mo_5_O_14_/C composite synthesized by sol-gel method was determined using thermal gravimetry (TG) and the differential scanning colorimetric (DSC) experiments. Figure 2b,c show the TG curves of the as-prepared P-MoO_3_ and the Mo_5_O_14_/C composite, respectively, recorded in the range 25–1000 °C in air. The thermal behavior of P-MoO_3_ does not show obvious weight loss until the decomposition of MoO_3_ (melting point at ca. 795 °C) [27]. The situation for the Mo5O14/C composite is different. The TG curve shows a strong endothermic peak at ~500 °C with a weigh loss of 26% corresponding to the evolution of CO_2_ through reaction between carbon in the composite and the atmospheric oxygen. Thus, the composite contains 26% carbon coming from the pyrolysis of citric acid. Upon further heating, the small exothermic peak appearing at ca. 800 °C is attributed to the melting of Mo_5_O_14_ in the composite similarly to the feature observed for pure MoO_3_ crystal. Further elemental determination of carbon was performed using a CHNOS analyzer that gave the same percentage of carbon (25.8 ± 0.2%) in the composite.

**Figure 3:**
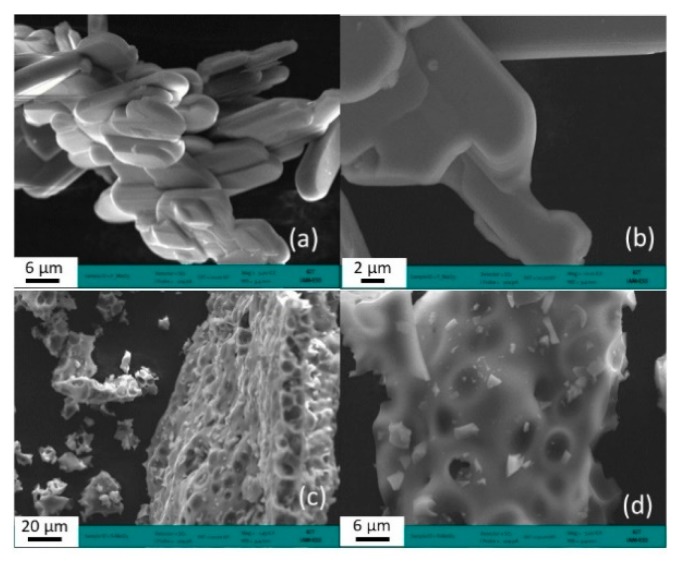
SEM images of P-MoO_3_ (**a**,**b**) and Mo_5_O_14_/C composite (**c**,**d**) synthesized by sol-gel method assisted by citric acid as chelating agent.

The morphology of the Mo_5_O_14_/C composite was analyzed by scanning electron microscopy and compared with that of pure MoO_3_ synthesized from the same precursor (Figure 3). While the SEM images (a,b) of P-MoO_3_ show uniformly distributed and well-developed packed platelets of the layered α-MoO_3_ structure, the SEM images (c,d) of the Mo_5_O_14_/C composite display a honeycomb carbon matrix with small pores, and irregular particles of molybdenum oxide are dispersed inside the matrix.

**Figure 4:**
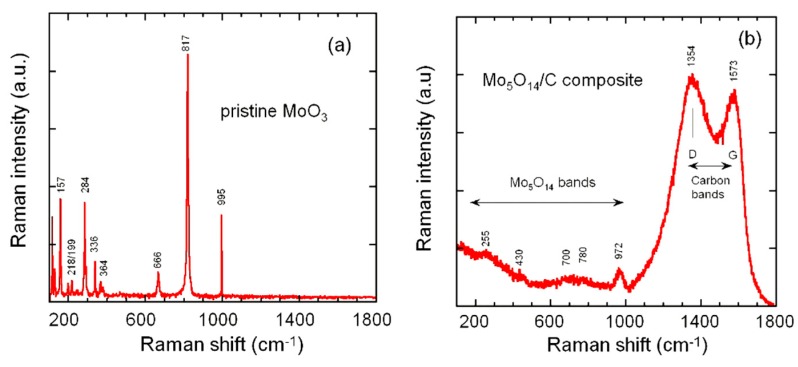
Raman spectra of the α-MoO_3_ (**a**) and Mo_5_O_14_/C composite (**b**) synthesized by sol-gel method assisted by citric acid as chelating agent. This spectrum was recorded at a spectral resolution of 2 cm^−1^ using the 514.5 nm laser line.

Since XRD experiments are not efficient tools to determine the structure of ill-crystallized materials, we investigated the short-range local structure of Mo_5_O_14_/C composite using Raman spectroscopy. Figure 4 compares the Raman patterns of the pristine MoO_3_ (a) and the Mo_5_O_14_/C composite (b). The Raman spectrum of Mo_5_O_14_/C composite prepared at 500 °C in argon atmosphere can be considered as the sum of the Mo_5_O_14_ and carbon contribution. In the low-frequency region, the spectrum shows features of the molybdenum oxide phase, while the high-frequency range (greater than 1200 cm^−1^) displays the fingerprint of the carbon matrix. Note that the weak intensity of Mo_5_O_14_ Raman pattern is due to the presence of carbon environment absorbing the scattering light. The five low-frequency Raman bands located at 972, 780, 700, 430 and 225 cm^−1^ can be identified as the vibrational patterns of tetragonal Mo_5_O_14_. The Mo-O stretching mode appears at 972 cm^−1^, the Mo-O-Mo bridging modes are recorded at 780, 700 and 430 cm^−1^, while the deformation modes of the Mo-O bonds appear at ca. 225 cm^−1^. This deformation mode was observed at 229 cm^−1^ in Mo_4_O_11_. It should be noted that this Raman spectrum definitely differs from those of either α-MoO_3_ (Figure 4a) or Mo_4_O_11_ phase [28,29]. For example, in the spectrum of Mo_5_O_14_, the broad peak at ~780 cm^−1^ replaces the sharp peak of α-MoO_3_ at 820 cm^−1^, which reveals the presence of sub-stoichiometric Mo_m_O_3m−1_ crystals. Such a blue shift of the stretching mode is attributed to the modification of Mo-O bonds induced by the reduction of Mo^6+^ to Mo^5+^ next to the oxygen vacancies [30]. Nazri and Julien [31] showed that the intensities and positions of vibrational bands of MoO_3_·nH_2_O are extremely sensitive to small changes of the Mo-O polyhedra. A loss of the translational symmetry is expected because of the oxygen vacancies present in the Mo_m_O_3m−1_ lattice. Thus, the highest stretching mode appears at 996 cm^−1^ in MoO_3_, while it is shifted to 985 cm^−1^ in Mo_4_O_11_. The Hardcastle–Wachs approach allows to correlate the Raman shifts with the Mo–O bond distances [32]. The stretching at 972 cm^−1^ observed in the Raman spectrum of Mo_5_O_14_ appears at the same frequency as that of MoO_3_·nH_2_O with a Mo-O distance of 1.687 Å against 1.671 Å for anhydrous α-MoO_3_. The broad bands observed in the range 1200–1600 cm^−1^ correspond to the D- and G-bands of carbon. The D-band at 1354 cm^−1^ is associated with the disorder-allowed A1g zone-edge mode of graphite, whereas the G-band centered at 1573 cm^−1^ corresponds to the optically allowed *E*_2g_ zone center mode of crystalline graphite. The broadening of the G- and D-bands are characteristics of localized in-plane *sp*^2^ domains, and disordered graphitic-like carbon, respectively. The peak intensity ratio *I*_D_/*I*_G_ of approximately 1.19 confirms the low degree of graphitization of the carbon matrix and the small size of the graphitic domains *L*_a_ of about 4.8 nm according to the empirical equation *L*_a_ (nm) = 4.4 *I*_D_/*I*_G_ [33]. The limited graphitization is due to the low temperature used for the preparation of the composite (i.e., 500 °C), but seems enough to be beneficial for electron transport from/to the semiconducting Mo_5_O_14_ active material.

### 3.2. BET-Surface Area

**Figure 5:**
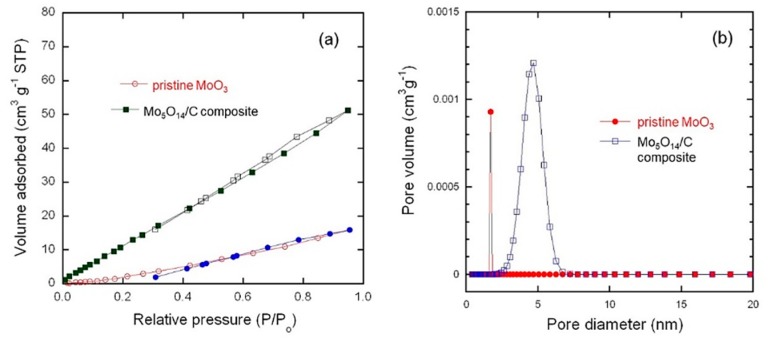
N_2_ adsorption-desorption isotherms (**a**) and pore size distribution (**b**) for P-MoO_3_ and Mo_5_O_14_/C composite.

Figure 5 shows the nitrogen adsorption–desorption isotherms and pore-size distributions (PSD) for both P-MoO_3_ and Mo_5_O_14_/C composite. The uptake of nitrogen adsorption of both samples increases gradually with the increase of relative pressure (Figure 5a). Note that the quantity of nitrogen adsorbed for the Mo_5_O_14_/C composite is much larger than that of P-MoO_3_ sample. The adsorption rises noticeably at low relative pressures of less than 0.5, indicating that micropores are present. Instead of reaching a plateau, the uptake of nitrogen further increases with relative pressure until *P*/*P*_0_ approaches unity, which suggests that a substantial amount of external surface area was created in Mo_5_O_14_/C sample. As shown in PSD curves (Figure 5b), the distribution of the pore diameters has peaks at 4.8 nm for Mo_5_O_14_/C composite. The Mo_5_O_14_/C composite exhibits a broader pore-size distribution with the pore diameter in the range 2−7 nm. It has been widely reported that physical and chemical activation of carbon materials help to open and widen the microporosity even with a conversion to mesopores [34,35]. The material porosity, i.e., pore size distribution and pore volume, has been quantitatively evaluated using the Barrett–Joyner–Halenda (BJH) method [36]. Results are listed in Table 1 and show that the BET surface area, the BJH total pore volume and the porosity are higher in Mo_5_O_14_/C than in P-MoO_3_. The increase of the specific surface area is attributed to the presence of 26 wt.% carbon. The higher pore size in the Mo_5_O_14_-type/C composite also favors better electrochemical performance of the electrode.

### 3.3. Electrochemical Properties

**Figure 6:**
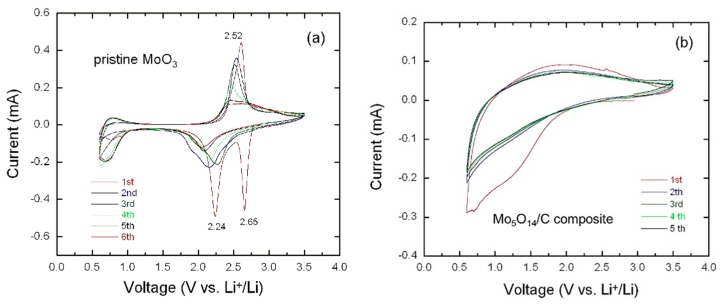
CV curves of the initial five cycles for P-MoO_3_ (**a**) and Mo_5_O_14_/C composite (**b**) recorded at the scanning rate of 0.05 mV s^−1^.

The cyclic voltammetry (CV) behavior for pristine MoO_3_ and Mo_5_O_14_/C composite electrodes performed in the potential range 0.6–3.5 V vs. Li^+^/Li at the scanning rate of 0.05 mV s^−1^ are displayed in Figure 6a,b, respectively. The choice of the low voltage cut-off of 0.6 V was intentional because of the formation of metallic Mo and 3Li_2_O, which takes place as a plateau at around 0.4–0.5 V upon further Li^+^ insertion [37,38]. The CV curves of pristine MoO_3_ exhibit well-defined redox peaks, due to the fact that this material is well-crystallized. In the first cycle, there are two strong cathodic peaks at ca. 2.7 and 2.3 V, with one reversible anodic peak around 2.6 V. The cathodic peak at 2.7 V is irreversible and disappeared after the first lithiation during subsequent cycles (Figure 6a). Such a behavior has been evidenced by preparing pre-lithiated Li_x_MoO_3_ samples using the chemical insertion by LiI in the range 0.1 ≤ *x* ≤ 1.0. For *x* ≈ 0.49, the CV curve displays only one reversible redox couple that is an indication of the disappearance of the pure α-MoO_3_ phase with similar patterns for subsequent cycles [39]. The mesoporous Mo_5_O_14_/C composite reveals a featureless voltammetry response over a broad potential range; this pattern arises from its amorphous-like structure, which leads to numerous insertion sites for Li^+^ ions. After the first cycle, a high reversibility is observed from overlapping CV curves (Figure 6b.)

**Figure 7:**
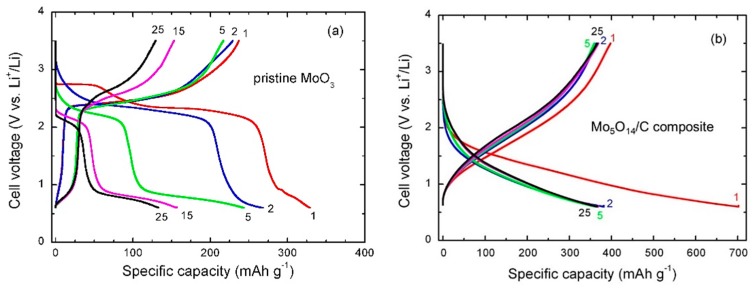
Typical galvanostatic charge-discharge curves of P-MoO_3_ (**a**) and Mo_5_O_14_/C composite (**b**) electrodes. Experiments were performed in 1 mol L^−1^ LiPF_6_/EC:DMC electrolyte at C/10 rate (70 mA g^−1^).

Figure 7a,b present the galvanostatic charge-discharge (GCD) profiles of the pristine MoO_3_ and Mo_5_O_14_/C composite electrodes, respectively, recorded at C/10 rate (70 mA g^−1^). In a fresh cell, pristine MoO_3_ and Mo_5_O_14_/C composite are electrodes in a “charged state” with all Mo ions with the 6+ oxidation state. Thus, the first process of lithiation occurs during discharge (reduction of Mo to 5+ and 4+ states) providing a capacity Q. During the subsequent charge Q’, Mo is oxidized, but not all Li ions can be extracted from the lithiated phase, thus less electrons are transferred and Q’<Q. Definitely, the electrochemical behaviors of these electrodes are different. The difference between the shape of charge/discharge curves of pristine MoO_3_ and Mo_5_O_14_/C is mainly due to the difference in the structure and morphology of the active material. In contrast with the 2D structure of α-MoO_3_, Mo_5_O_14_ has a 3D structure with empty site in the tunnels inducing a different Gibbs energy that results in unlike voltage profile. In addition, the voltage profile of an amorphous material is a S-shape curve due to the random distribution of the available site in the framework.

**Figure 8:**
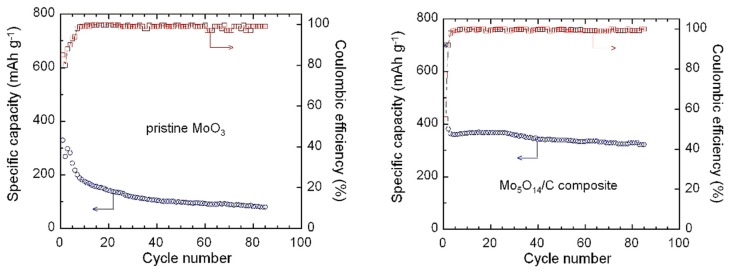
Cycling performance of pristine MoO_3_ and Mo_5_O_14_/C composite electrodes. Cells were tested at C/10 rate using 1 mol L^−1^ LiPF_6_/EC:DMC electrolyte.

**Figure 9:**
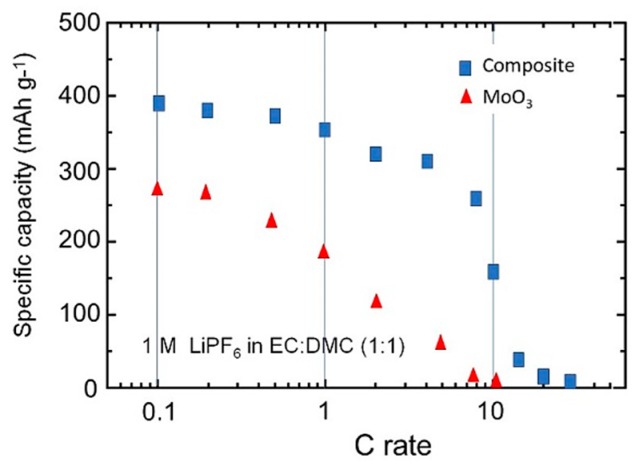
Rate capability of pristine MoO_3_ and Mo_5_O_14_/C composite electrodes.

In the first discharge process, the crystallized pristine MoO_3_ shows two typical plateaus (Figure 7a), in accordance with the cathodic peaks in the CV curve (Figure 6a). The insertion of lithium proceeds in two steps at 2.75 and 2.30 V vs. Li^+^/Li within the capacity range of 0–50 and 100–250 mAh g^−1^, respectively. The disappearance of the first plateau at the second cycle is related to an irreversible structural change, suggesting that part of Li^+^ ions cannot be extracted during the charge process. This structural modification has been described by the phase transition from MoO_3_ to Li_x_MoO_3_ (where *x* ≤ 0.25), which induces a pronounced expansion of the interlayer spacing (from 0.69 to 1.175 nm) [39,40]. Similar behavior was previously observed for lithium intercalation in the layered V_2_O_5_ host [41]. The specific capacity of the P-MoO_3_ electrode decreases continuously over subsequent discharge-charge cycles from 330 mAh g^−1^ (1st cycle) to 136 mAh g^−1^ (25th cycle). This large capacity loss (2% per cycle) reveals the poor electrochemical stability of the α-MoO_3_ phase prepared by citrate-gel method. In contrast, the mesoporous Mo_5_O_14_/carbon composite exhibits better electrochemical performance. GCD curves are featureless (without plateau) showing a steady decay of the cell voltage upon Li insertion. Such discharge-charge profiles are characteristic of an amorphous phase, which implies a wide variation in energy for the Li sites available for insertion [42,43]. For the first discharge, this electrode delivers a specific capacity of 703 mAh g^−1^, decreasing to 388 mAh g^−1^ at the 2nd cycle. During next cycles, the discharge capacities slightly decrease to reach 325 mAh g^−1^ at the 50th cycle. The cyclability of pristine MoO_3_ and Mo_5_O_14_/C composite electrodes was tested at C/10 rate in the cell voltage range of 0.6–3.5 V as shown in Figure 8. The Mo_5_O_14_/C composite shows an almost stable electrochemical behavior up to 85 cycles with a rate of capacity fade of 0.8% per cycle. Results of the rate capability are presented in Figure 9. The high performance of the Mo_5_O_14_/C composite is clearly revealed with a specific discharge capacity of 155 mAh g^−1^ at 10C rate (7 A g^−1^).

## 4. Discussion

The structural and electrochemical properties of a composite formed of oxygen-deficient Mo_m_O_3m−1_ and carbon have been examined for the first time. Mo_m_O_3m−1_ compounds are particularly interesting, mainly due to the structure with large tunnels accessible for Li^+^ ions. In addition, they have a high electrical resistivity, i.e., *ρ* = 1 Ω cm for Mo_8_O_23_ and *ρ* = 78 Ω cm^−1^ for Mo_18_O_52_ that are semiconductors, while η-Mo_4_O_11_ is metallic with *ρ* = 1.7 10^−4^ Ω cm [44]. In the work presented here, the reduction of the layered structure MoO_3_ obtained by the decomposition of citric acid as chelating agent and ammonium molybdate tetrahydrate as raw material has been performed in closed atmosphere. The as-prepared mesoporous Mo_5_O_14_/C composite was successfully obtained by a moderate annealing temperature of 500 °C. This new material is composed of a honeycomb carbon matrix, in which amorphous Mo_5_O_14_ nanoparticles are introduced. It is assumed that the Mo^6+^ ions were reduced by the presence of CO, CO_2_ and NH_3_ gases coming from the chelate and raw material forming the non-stoichiometric (oxygen-deficient) Mo_5_O_14_/C compound. This tunnel-like structure accommodates oxygen deficiency via oxygen vacancies and crystallographic shear planes. The formation of an intermediate compound Mo_m_O_3m−1_ between Mo^VI^O_3_ and Mo^IV^O_2_ during the reduction of MoO_3_ has been reported many times [15,45,46], but here, despite the previous claims, we obtained a stable phase with the O/Mo ratio of 2.8.

Also, this work illustrates the synergy between a molybdenum oxide and carbon that exhibits excellent electrochemical performance of the composite. Also, it reveals the importance of electronic conductivity and the mesoporous nature for substantial lithium insertion in tunnel-like molybdenum-oxide compounds. In contrast to the large capacity fade due to the interlayer spacing expansion in crystalline α-MoO_3_, the excellent electrochemical behavior of the Mo_5_O_14_/C composite electrode is due to the formation of large mesopores. The unique cycling performance of the composite is attributed to the presence of carbon, which plays a dual role in enwrapping the tunnel-like Mo_5_O_14_ nanoparticles and accommodating the volume change. Graphitized carbon provides a large contact surface and acts as an excellent conductive agent facilitating the charge-discharge process. The porosity formed by carbon facilitates the transport of charge carriers and the delocalized insertion site in the amorphous matrix enhances the kinetics of Li^+^ ions. According to its high reversible capacity and good cycling stability, the Mo_5_O_14_/C composite is a promising alternative electrode material for application in lithium-ion batteries.

Several studies have demonstrated the improved cycling performance of oxygen-deficient Mo_m_O_3m−1_ compounds compared with stoichiometric MoO_3_. In an early work, Besenhard and Schöllhorn [4] have shown that discharge-charge reactions of Mo_18_O_52_ and Mo_8_O_23_ electrodes in organic Li^+^ electrolytes occur via reversible topotactic redox processes. Cignini et al. examined the performance of various non-stoichiometric Mo_m_O_3m−1_ compounds as cathodes in Li cells, i.e., Mo_8_O_23_, Mo_9_O_26_, Mo_17_O_47_ and Mo_4_O_11_ and showed that Mo_5_O_14_ can deliver a specific capacity of 310 mA g^−1^ when discharged at 0.5 mA cm^−2^ in the voltage range 2.5–1.0 V [5]. Christian and co-workers [9] reported that for the oxides Mo_4_O_11_ (MoO_2.75_), Mo_17_O_47_ (MoO_2.765_), Mo_8_O_23_ (MoO_2.889_), and Mo_9_O_26_ (MoO_2.875_) the highest reversible capacity (1.5Li/Mo) was obtained for Mo_17_O_47_ after several deep discharge-charge cycles in the potential range 2.9–1.4 V. The reversible capacities of other Mo_m_O_3m−1_ compounds are considerably less than that of Mo_17_O_47_ except for Mo_4_O_11_, which exhibits a reversible capacity of 0.75Li/Mo after 20 deep cycles. These results suggest that both channel/site size and electronic conductivity are the predominant factors influencing the extent of reversible Li insertion by Mo_m_O_3m−1_ oxides with tunneled framework. Distortions and nonequivalence of the available sites should also affect the thermodynamics of the reduction processes. Julien et al. showed that the first discharge profile of a crystalline Li_x_MoO_2.8_ (lithiated Mo_5_O_14_) displays a stepped behavior with a voltage plateau at ca. 2.2 V followed by a potential decline for *x* > 0.7Li/Mo and a total Li uptake of 1.45Li/Mo for a cut-off voltage of 1.0 V. For all Mo ions reduced to Mo^4+^, the expected lithium uptake would be 8/5(1.6) Li/Mo; a value close to 258 mAh g^−1^ measured by electrochemical titration [11]. Jung et al. [14] showed that during the first discharge, MoO_2.895_ prepared by ball milling delivered a specific of ca. 600 mAh g^−1^ that decreased to 420 mAh g^−1^ (2nd cycle) in the voltage range 3.5–0.5 V. For comparison, electrochemical properties of composites formed by a Mo oxide and a carbonaceous material are listed in Table 2.

According to the GCD profile presented in Figure 7b, the initial discharge capacity and the initial Coulombic efficiency of the Mo_5_O_14_/C composite are 703 mAh g^−1^ and 55%, respectively. In the same voltage range, a composite electrode made of MoO_3_ nanocrystals distributed in an amorphous carbon matrix delivered a specific capacity of approximately 630 mAh g^−1^ and an initial Coulombic efficiency of 75% [47]. An initial discharge capacity of approximately 550 mAh g^−1^ with a Coulombic efficiency of 69% was reported for crumpled graphene–MoO_3_ composite cycled at a constant current density of 2 A g^−1^ [22]. Furthermore, the higher performance of the Mo_5_O_14_-type/C composite electrode is attributed to the accommodated volume expansion. Christian et al. [9] have shown that the unit cell of the fully lithiated Mo_5_O_14_ crystal expands in the *b* direction and contracts in both the *a* and *c* directions upon insertion of lithium. Also, we believe that the presence of 26 wt.% carbon homogeneously distributed and associated with the intrinsic properties of the active particles minimizes the volume expansion.

## 5. Conclusions

Pure α-MoO_3_ and mesoporous Mo_5_O_14_-type/C composite were successfully prepared by a citrate–gel method from ammonium molybdate precursor. Crystalline orthorhombic α-MoO_3_ is observed for xerogel calcined in air, while the Mo_5_O_14_/C composite is prepared under argon atmosphere. The latter one is a poorly crystalline material that is composed of Mo_5_O_14_ nanograins incorporated into a carbon matrix as noticed from X-ray diffraction and Raman spectroscopy. The carbon content of approximately 26 wt.% was estimated from TGA and elemental analysis. Analysis of the vibrational features of D- and G- Raman bands of the carbon reveals that it is moderately graphitized. BET measurements show that the Mo_5_O_14_/C composite exhibits a surface area of 7.1 m^2^ g^−1^, average pore size of approximately 4 nm and total pore volume of 0.07 cm^3^ g^−1^, which are favorable for enhanced electrochemical insertion of Li^+^ ions into the mesoporous lattice.

The Mo_5_O_14_/C composite appears to be electrochemically stable showing a specific capacity of 325 mAh g^−1^ and a capacity fade of 0.8% after 50 cycles at 0.1C. Such a good performance is attributed to the mesoporous nature of the electrode material and the presence of carbon, which suppresses the aggregation of molybdenum-oxide particles and thus increases their structural stability during cycling, while keeping the particles electrically connected.

## Figures and Tables

**Table 1:** Results of the BET experiments P-MoO_3_ and Mo_5_O_14_-type/C composite.

Sample	BET Surface Area (m^2^ g^−1^)	Total Pore Volume (cm^3^ g^−1^)	Average Pore Size (nm)
P-MoO_3_	4.5	0.0024	2.19
Mo_5_O_14_-type/C	7.1	0.0719	4.01

**Table 2:** Electrochemical properties of composites formed by Mo oxides and carbonaceous materials. Initial discharge specific capacities are obtained at a cut-off of 0.6 V vs. Li^+^/Li.

Composite ^(a)^	Cathode Loading (mg cm^−2^)	Specific Capacity (mAh g^−1^)	Current Rate	Ref.
MoO_3_/a-C	1.5	630	1 A g^−1^	[47]
MoO_3_/CNTs ^(b)^	-	300	C/5	[48]
MoO_3_/C	-	~600	40 mA g^−1^	[18]
Mo_3_O_8_/CNTs	-	~450	80 mA g^−1^	[49]
Carbon-free MMO ^(c)^	-	300	0.1 A g^−1^	[50]
MoO_3_/C	-	~420	0.2C	[23]
MoO_3_/27 wt.% Gr	-	~450	50 mA g^−1^	[17]
MoO_3_/47 wt.% Gr	-	~480	50 mA g^−1^	[17]
MoO_3_/C	1.5	~350	0.1 A g^−1^	[19]
MoO_3_/Gr	1.2	~550	2 A g^−1^	[22]
Mo_5_O_14_/C	2.1	703	C/10	this work

^(a)^ CNTs: carbon nanotubes; rGO: reduced graphene oxide; Gr: graphene; a-C: amorphous carbon. ^(b)^ Voltage range of 3.5–1.5 V vs. Li^+^/Li; ^(c)^ MMO (MoO_2.63_) is a mixture of MoO_3_+MoO_2_+Mo_4_O_11_.
